# Occurrence of Yeasts in White-Brined Cheeses: Methodologies for Identification, Spoilage Potential and Good Manufacturing Practices

**DOI:** 10.3389/fmicb.2020.582778

**Published:** 2020-10-15

**Authors:** Athina Geronikou, Thanyaporn Srimahaeak, Kalliopi Rantsiou, Georgios Triantafillidis, Nadja Larsen, Lene Jespersen

**Affiliations:** ^1^Department of Food Science, Faculty of Science, University of Copenhagen, Frederiksberg, Denmark; ^2^Department of Agricultural, Forestry and Food Sciences, University of Turin, Turin, Italy; ^3^Jotis S.A., Food Industry, Athens, Greece

**Keywords:** white-brined cheese, spoilage yeasts, yeast identification, off-flavors, microbial interactions, GMP

## Abstract

Yeasts are generally recognized as contaminants in the production of white-brined cheeses, such as Feta and Feta-type cheeses. The most predominant yeasts species are *Debaryomyces hansenii*, *Geotrichum candidum*, *Kluyveromyces marxianus*, *Kluyveromyces lactis*, *Rhodotorula mucilaginosa*, and *Trichosporon* spp. Although their spoilage potential varies at both species and strain levels, yeasts will, in case of excessive growth, present a microbiological hazard, effecting cheese quality. To evaluate the hazard and trace routes of contamination, the exact taxonomic classification of yeasts is required. Today, identification of dairy yeasts is mainly based on DNA sequencing, various genotyping techniques, and, to some extent, advanced phenotypic identification technologies. Even though these technologies are state of the art at the scientific level, they are only hardly implemented at the industrial level. Quality defects, caused by yeasts in white-brined cheese, are mainly linked to enzymatic activities and metabolism of fermentable carbohydrates, leading to production of metabolites (CO_2_, fatty acids, volatile compounds, amino acids, sulfur compounds, etc.) and resulting in off-flavors, texture softening, discoloration, and swelling of cheese packages. The proliferation of spoilage yeast depends on maturation and storage conditions at each specific dairy, product characteristics, nutrients availability, and interactions with the co-existing microorganisms. To prevent and control yeast contamination, different strategies based on the principles of HACCP and Good Manufacturing Practice (GMP) have been introduced in white-brined cheese production. These strategies include milk pasteurization, refrigeration, hygienic sanitation, air filtration, as well as aseptic and modified atmosphere packaging. Though a lot of research has been dedicated to yeasts in dairy products, the role of yeast contaminants, specifically in white-brined cheeses, is still insufficiently understood. This review aims to summarize the current knowledge on the identification of contaminant yeasts in white-brined cheeses, their occurrence and spoilage potential related to different varieties of white-brined cheeses, their interactions with other microorganisms, as well as guidelines used by dairies to prevent cheese contamination.

## Introduction

Cheese making, particularly of white-brined cheeses, is one of the oldest dairy technologies, originated from the Mediterranean region and the Middle East more than 8000 years ago. Most recent scientific literature has been dedicated to white-brined cheeses produced in the Balkan Peninsula region, Turkey, Northern Africa, European countries, and some parts of Asia and Latin America (Hayaloglu, 2017). Today, many varieties of white-brined cheeses, with typical representatives being Feta and Feta-type cheeses, are produced and consumed worldwide. Only in Greece, production of Feta exceeds 110,000 tons per year (Hellenic Statistical Authority, 2018). White-brined cheeses are made from goat, sheep or cow milk, or a mixture of milks, and characterized by a creamy smooth texture and a mild salty and/or acidic taste. Traditionally, Feta-type cheeses were solely made from raw milk, but, nowadays, industrial dairies pasteurize the milk and use commercial starter cultures. Manufacture of the white-brined cheeses includes milk pasteurization, addition of the starter cultures and rennet, followed by milk coagulation and curd formation. Afterwards, curd is drained in molds, cut into pieces, salted (dry or in brine) and ripened in brine for typically several months (Hayaloglu, 2017).

Yeasts are widely spread in dairy production and frequently found in raw milk, brine, air, production surfaces, cheese vat and cloth, curd cutting knife, etc. (Sharaf et al., 2014; Banjara et al., 2015). In some types of cheeses, such as blue-veined and smear-ripened cheese, yeasts can be used as adjunct cultures, affecting the ripening process, formation of aroma compounds, and interaction with starter cultures (Ferreira and Viljoen, 2003; Kesenkaş and Akbulut, 2008; Gori et al., 2013; Ryssel et al., 2015). In white-brined cheeses, yeasts are not included as starter cultures and frequently referred as contaminants, though, sometimes, they are a part of secondary microflora (Kesenkaş and Akbulut, 2008). The most frequent yeast species in white-brined cheeses are *Debaryomyces hansenii*, *Geotrichum candidum*, *Kluyveromyces lactis*, *Kluyveromyces marxianus*, *Saccharomyces cerevisiae*, *Trichosporon cutaneum*, *Yarrowia lipolytica*, and *Candida* spp. (Golić et al., 2013; Karasu-Yalcin et al., 2017). It should be noted, that *G. candidum* was formerly recognized as the anamorph form of *Galactomyces geotrichum* (De Hoog et al., 1998) and, as such, both species names have been used more or less randomly in dairy literature. Based on later studies, the species *Galactomyces candidus* was accepted as the teleomorph form of *G. candidum*, and *G. geotrichum* was accepted as a separate species (De Hoog and Smith, 2004, 2011), which might cause some confusion referring especially to older literature. As most dairy isolates seem to belong to *G. candidum*, as characterized by Perkins et al. (2020), in the current review the species name *G. candidum* is generally used unless *G. geotrichum* is explicitly mentioned in the original literature.

Yeast spoilage activities might lead to alteration of the organoleptic properties, decreased shelf life, and impaired quality of the dairy products (Salustiano et al., 2003; Radha and Nath, 2014). Yeast propagation in dairy products, particularly in white-brined cheeses, depends on numerous factors, such as the composition of milk, nutrients availability, interactions with co-existing microorganisms, production, and storage conditions (Soliman and Aly, 2011; Buehler et al., 2017; Laèanin et al., 2017). Furthermore, it is well known that yeasts differ in their biochemical functions and metabolic activities and, consequently, in their spoilage behavior (Akabanda et al., 2013; Haastrup et al., 2018; Bayili et al., 2019). In this context, accurate taxonomic identification of yeast species and yeast genotyping to the strain level are essential to evaluate the spoilage potential of any yeast contaminants. However, despite the importance of yeasts as spoilage microorganisms in white-brined cheeses, limited knowledge exists on the spoilage potential of the different yeast species and the specific traits, leading to their quality defects. The aim of the present review is, therefore, to give an overview on state-of-the-art technologies for identification and detection of spoilage yeasts, the diversity of yeast species able to cause quality defects in white-brined cheeses, extrinsic and intrinsic factors influencing their spoilage potential, the role of bio-protective cultures and other microbial interactions, as well as the routes of contamination and good manufacturing practices.

## Identification and Detection of Yeasts in Dairy Products

### Conventional and Molecular-Biological Methodologies for Species Identification

Traditional methods of yeast identification in dairy products are based on macro- and micro-morphological observations and physiological characteristics, such as growth requirements, assimilation, and fermentation of carbohydrates and nitrogen (Garnier et al., 2017a). Phenotypic indicators, however, are highly heterogeneous and uncertain, as different yeast species might exhibit close morphological and physiological features. Traditional culturing techniques are commonly combined with molecular approaches to identify yeast species, associated with different types of dairy products and production environments. Currently, sequencing of the D1/D2 region of the 26S rRNA gene and the internal transcribed spacer (ITS) domains (ITS1 and ITS2) divided by the conserved 5.8S rRNA gene, are the most frequently used molecular methods of yeast identification (Lopandic et al., 2006; Gori et al., 2013; Buehler et al., 2017; Garnier et al., 2017b; Haastrup et al., 2018; Tokak et al., 2019; Merchán et al., 2020). Both approaches employ extensive databases to ensure discrimination between closely related yeast species. For instance, Garnier et al. (2017b) performed 26S rRNA gene sequencing to classify spoilage yeasts to species level, e.g., *Candida inconspicua*, *Candida intermedia*, *Candida parapsilosis*, *D. hansenii*, *G. candidum*, *K. lactis*, *K. marxianus*, *Meyerozyma guilliermondii*, *Pichia fermentans*, and *Y. lipolytica*, from French dairy products (cream, fresh cheese, smear cheese, etc.) and factory air. Application of ITS sequencing in another extensive study (346 fungal dairy isolates), revealed that species within the genera *Debaryomyces*, *Candida*, and *Kluyveromyces* were dominating in raw and pasteurized milk cheeses (Buehler et al., 2017).

Recently, an enhanced focus has been directed toward specific phenotypic identification techniques, such as matrix-assisted laser desorption ionization-time of flight mass spectrometry (MALDI-TOF MS) and Fourier transform infrared spectroscopy (FTIR), enabling rapid and cost-effective taxonomic identification of dairy-associated yeasts. MALDI-TOF MS generates protein-based spectral profiles (fingerprints) acquired by desorption of specific peptide/protein biomarkers released from the cell surface by acidic treatment (Pinto et al., 2011; Chalupová et al., 2014). This method was successfully applied for the identification of contaminating yeast species in yogurts and cheeses (Kačániová et al., 2018; Halil Kılıç, 2019). FTIR is based on the detection of functional biochemical groups directly from intact cells, producing metabolic spectral “fingerprints” unique for yeast species (Wenning et al., 2002; Büchl et al., 2008; Patel, 2019). Identification of a given yeast isolate from a fingerprint-like spectrum requires a comprehensive reference database. Previously, especially the MALDI-TOF MS databases comprised predominantly clinical isolates, which presented a notable limitation for typing of the dairy-related yeasts (Dongowski et al., 2000; Larpin-Laborde et al., 2011; Moothoo-Padayachie et al., 2013). It is acknowledged, however, that the databases are being gradually updated to cover a broader range of food-related yeast species. The drawback of FTIR is the high sensitivity to growth conditions of yeasts and the sample preparation procedure, which, together with insufficient database size, might lead to poor accuracy of yeast identification (Colabella et al., 2017).

### Molecular-Biological Methodologies for Identification at the Strain Level

Strain typing is essential to trace the yeasts “hot spots” in dairy production in order to prevent contamination and extend the dairy shelf life. Various genotyping or DNA fingerprinting techniques are currently applied for the identification of dairy yeasts at species and strain levels. For screening purposes, yeast genotyping and cluster analysis of the DNA fingerprints are often introduced prior DNA sequencing. As a standard strain-typing approach, pulsed-field gel electrophoresis (PFGE) is used to evaluate intraspecies diversity of chromosome arrangements or chromosome-length polymorphism (Miller, 2013; Lopez-Canovas et al., 2019). The DNA separation by PFGE relies on the ability of intact yeast chromosomes to reorient and migrate in a new direction in agarose gel in response to an alternating electric field. High discrimination power, robustness, and reproducibility of PFGE were verified for *D. hansenii* strains collected from the production of Danish surface−ripened cheeses (Petersen and Jespersen, 2004), the dairy strains of *K. marxianus* (Fasoli et al., 2015; Naumova et al., 2017), and *S. cerevisiae* (Hage and Houseley, 2013).

Other genotyping techniques, allowing to differentiate closely related yeast species up to the strain level, are based on the PCR amplification with the use of primers, targeting repeated DNA sequences along the chromosome (Lopandic et al., 2006; Gori et al., 2013). Among them, the 5′-anchored primer (GTG)_5_ repetitive-PCR fingerprinting was applied, e.g., for characterization of yeast communities in soft cheese from Spain (Merchán et al., 2020), Danish surface-ripened cheeses (Gori et al., 2013), and spontaneously fermented milk products from West Africa (Akabanda et al., 2013; Bayili et al., 2019). Randomly amplified polymorphic DNA (RAPD), employing a single primer M13 for random amplification of complementary genome sequences, was used for taxonomic classification of yeasts in Fiore Sardo cheese (hard cheese from raw sheep’s milk) (Fadda et al., 2004), fresh and sour curd cheeses (Lopandic et al., 2006), variety of Italian and Greek cheeses (Mozzarella, Caprino, Feta, etc.) (Andrighetto et al., 2000), and for differentiation of *D. hansenii* strains isolated from raw milk cheeses (Padilla et al., 2014).

Mitochondrial DNA (mtDNA) restriction fragment length polymorphism (RFLP) analysis is another common yeast typing method, which involves PCR amplification of the 5.8S-ITS rDNA regions, followed by digestion with two or more restriction enzymes, typically *Cfo*I, *Hae*III, *Hpa*II or *Hin*fI. The RFLP of the ITS regions was efficiently applied for typing of *D. hansenii* strains, isolated from the surface-ripened cow’s milk cheeses of the Danbo type (Petersen et al., 2002) and the Gubbeen Farmhouse cheese (Mounier et al., 2006), and for characterization of yeast microbiota, composed of *D. hansenii*, *K. marxianus*, *K. lactis*, *M. guilliermondii*, *Y. lipolytica*, *Trichosporon coremiiforme*, *Trichosporon domesticum*, and *Candida* spp., in goat and ewe’s milk cheeses (Padilla et al., 2014).

Multilocus sequence typing (MLST) is originally based on the analysis of polymorphic sites in a number of conserved housekeeping genes, serving as unique genetic markers (alleles) within a species (Muñoz et al., 2014). Advances in the whole genome sequencing allowed to optimize MLST schemes for specific species and increase discriminative power by scattering multiple loci within a genome. According to Tittarelli et al. (2018), MLST based on five housekeeping genes (*IPP1*, *TFC1*, *GPH1*, *GSY2*, and *SGA1*) provided sufficient polymorphic sites for classification and selection of *K. marxianus* strains in Italian cheeses. Lavoie et al. (2012) performed MLST using six loci (*ADE2*, *HIS3*, *LEU2*, *LYS2*, *NMT1*, and *TRP1*) to study the occurrence of *Issatchenkia orientalis* isolates from raw milk cheese. In a recent study, Perkins et al. (2020) applied the whole genome sequence approach to elucidate genetic diversity and evolutionary pathways of *G. candidum* isolated from smear-ripened cheeses and other sources. The authors developed a new MLST scheme based on six targeted loci (*ALA1*, *CDC19*, *SAPT4*, *GLN4*, *PGI1*, and *PGM2*) and identified 15 sequence types (STs) out of 41 strains, conferring that the allele variation arose from recombination events. The authors suggested that recombination events induced an adaptive divergence between the wild strains and the cheese-making strains of *G. candidum*.

### Novel Culture-Independent “Omics” Technologies

Recent advances in next-generation DNA sequencing (NGS) and bioinformatics tools have been adopted by the dairy sector to achieve deeper insights into diversity, succession, and interactions within microbial communities in dairy products. The NGS technologies allow high throughput sequencing of total microbial DNA or RNA without any prior culturing. Generally, the workflow includes DNA library preparation by amplification of a fragment of interest, using primers with indexing adapters specific to each platform, as previously described in many excellent reviews [recently reviewed by Kumar et al. (2019)]. In dairy studies, the DNA amplicons typically target 16S rRNA gene for lactic acid bacteria and 26S rRNA or ITS genes for yeasts. In earlier studies, the 454 pyrosequencing technology (Life Sciences, Roche) was employed, e.g., to investigate microbial succession during ripening of semi-hard Danbo cheeses (Ryssel et al., 2015) and co-occurrence of potential yeast spoilers and LAB in different types of cheese and production environment (Stellato et al., 2015). Currently, the rapidly evolving NGS Illumina technology established cost-effective and accurate DNA sequencing platforms (e.g., MiSeq and NextSeq 550), generating reads of more than 300 bp, i.e., compatible to the fragments recommended for yeast identifications by ITS1-5.8S-ITS2 rDNA (Schoch et al., 2012). Especially for scientific purposes, NGS Illumina technology is being a routinely used method to examine ecology and dynamics of microbiotas in diverse fermented milk products (Jatmiko et al., 2019; Sessou et al., 2019). For example, Ceugniez et al. (2017) described the evolution of yeasts during ripening of the Tomme d’Orchies type cheeses (France), in which prevalent species, such as *Y. lipolytica*, *G. geotrichum*, *Kluyveromyces* spp., and *Debaryomyces* spp., were detected by ITS2 rRNA gene sequencing (Illumina). NexSeq sequencing targeting ITS2, together with culture-dependent 26S rRNA gene sequencing, were applied to get insight into halotolerant yeast population in the brine of semi-hard Danbo cheese (Haastrup et al., 2018). A good correlation between culture-dependent and -independent techniques was observed for the predominant microorganisms (e.g., *Debaryomyces* spp., *Candida* spp., and *Yamadazyma* spp.). For less abundant yeast species (e.g., *Trichosporon* spp.), a lower correlation was observed due to the fact that both viable and dead cells could be detected by the NGS sequencing, differences in the DNA amplicons, isolation medium, etc. (Haastrup et al., 2018). Bertuzzi et al. (2018) applied whole-metagenome shotgun sequencing to screen the microbial population on the smear-ripened cheeses and analyzed the association of metagenomic clusters with the variation of pH, color, and flavor development. Using correlation analysis, it was possible to associate individual microorganisms with volatile compounds in the cheese surface. Among them, specifically, *D. hansenii* correlated with the production of alcohols and carboxylic acids, while *G. candidum* correlated with sulfur compounds (Bertuzzi et al., 2018).

In 2014, Oxford Nanopore Technologies (ONT) released a 3rd generation portative low-cost platform for DNA and RNA sequencing. Nanopore sequencers (MinION, GridION) measure the ionic current fluctuations, when single-stranded nucleic acids pass through protein-based nanopores. Compared to Illumina sequencing, ONT generates ultra-long DNA reads and eliminates amplification bias as no amplification step is needed for library construction (Amarasinghe et al., 2020). Nanopore long-read technology (MinION) has already been tested for *de novo* sequencing and assembling of the 21 strains of *S. cerevisiae* (Istace et al., 2017) and for identification of *S. cerevisiae*, *Rhodotorula graminis*, and *Malassezia* spp. (D’Andreano et al., 2020). Recent studies of microbiota from fresh and ripened cheeses demonstrated that the contiguity of microbial genome assemblies using shotgun MinION was much higher than the Illumina-only assemblies, allowing complete mapping of genes (e.g., transposable elements), which are generally missed using a short-read sequencing strategy (Ianni et al., 2020).

## Spoilage Yeast Species in White-Brined Cheeses

### Major Yeast Genera Occurring in White-Brined Cheeses

Depending on variety of white-brined cheeses, yeasts might comprise a part of the microflora, e.g., in a smear-forming surface layer, which may positively influence cheese flavor and texture without leading to quality defects (Bintsis et al., 2000). Occurrence of yeast species in white-brined cheeses within the genera *Debaryomyces*, *Geotrichum*, *Kluyveromyces*, *Pichia*, *Rhodotorula*, *Saccharomyces*, *Torulaspora*, *Trichosporon*, and *Yarrowia* has frequently been reported (Rantsiou et al., 2008; Golić et al., 2013; Cardoso et al., 2015; Šuranská et al., 2016; Karasu-Yalcin et al., 2017), however, the detailed information on yeast taxonomy, properties, and spoilage potential is often missing. Species *D. hansenii* is most frequently isolated from the cheese environment, most likely, due to its halophilic nature and affiliation to cheese brine (Haastrup et al., 2018). A reason for lacking an overview of spoilage yeasts in white-brined cheeses lies in an extensive number of artisanal products, which are traditionally produced by the herders with different production technologies around the world. Yeast contamination, specifically in artisanal products, is difficult to control. Diversity of the spoilage consortia and yeast propagation will typically vary between the dairies due to the differences in raw materials, usage of pasteurization, rennetting temperature (30–38°C) and time (40–180 min), brining conditions and salt concentration (7–16% NaCl), ripening temperature and storage period, the standards of hygiene during cheese making, etc. (Hayaloglu, 2017). The major varieties of white-brined cheeses and the associated potential spoilage yeasts are presented in Table 1.

**TABLE 1:** Occurrence of spoilage yeasts in white-brined cheeses.

Product	Cheese Category	Raw material (milk)	Yeast species	Country	References
Feta	Soft	Ovine or a mixture of ovine with caprine	*Candida sphaerica Candida zeylanoides Debaryomyces hansenii Kluyveromyces lactis Lachancea thermotolerans Pichia fermentans Pichia membranifaciens Tetrapisispora blattae Saccharomyces cerevisiae*	Greece	Vivier et al., 1994; Rantsiou et al., 2008; Litopoulou-Tzanetaki and Tzanetakis, 2011
Halloumi	Semi-hard	Ovine, caprine or mixture of them (optionally cow)	*Candida* spp*. Debaryomyces hansenii Pichia membranifaciens*	Greece, Cyprus	Kaminarides et al., 2009; Mehyar et al., 2018; Kamilari et al., 2020
White cheese	Soft	Cow	*Candida aaseri Candida boidinii Candida guilliermondii Candida intermedia Candida sake Candida zeylanoides Debaryomyces hansenii Geotrichum candidum Kluyveromyces lactis Kluyveromyces marxianus Naumovozyma dairenesis Pichia membranifaciens Torulaspora delbrueckii Yarrowia lipolytica*	Denmark	Westall and Filtenborg, 1998
White-pickled	Soft	Ovine, caprine or bovine	*Candida zeylanoides Cryptococus albidus Debaryomyces hansenii Filobasidium globisporum Galactomyces geotrichum Hanseniaspora uvarum Kluyveromyces lactis Torulaspora delbrueckii Torulaspora quercuum Trichosporon gracile Trichosporon ovoides Yarrowia lipolytica*	Serbia	Golić et al., 2013; Šuranská et al., 2016
White-brined	Unknown	Cow	*Candida* spp*. Rhodotorula* spp*. Saccharomyces* spp.	Bulgaria	Chipilev et al., 2016
Akawi	Semi-hard	Ovine, caprine or mixture of them	*Candida guilliermondii Debaryomyces hansenii*	Lebanon, Syria	Pachlová et al., 2016
Beyaz peynir (Turkish White cheese)	Semi-hard	Ovine, caprine, cow or mixture of them	*Candida* spp*. Candida zeylanoides Debaryomyces hansenii Kluyveromyces lactis Starmera amethionina Torulaspora delbrueckii*	Turkey	Öztürk and Şahin, 2000; Togay et al., 2020
Mihaliç	Hard	Ovine or caprine	*Candida bertae Candida catenulata Candida cylindracea Candida famata Candida inconspicua Candida krusei Candida paludigena Candida robusta Candida tropicalis Candida zeylanoides Clavispora lusitaniae Geotrichum candidum Kodamaea ohmeri Trichosporon asahii*	Turkey	Solak and Akin, 2013; Karasu-Yalcin et al., 2017; Togay et al., 2020
Jiben-Al-Arab	Soft	Ovine	*Candida albicans Candida krusei Candida parapsilosis Candida tropicalis Geotrichum candidum Rhodotorulla* spp.	Iran	Khalil et al., 2018
Domiati	Soft	Bovine	*Candida albicans Candida krusei Debaryomyces hansenii*	Egypt	Sharaf et al., 2014; Hameed, 2016
Serro Minas	Semi-hard	Bovine	*Candida atlantica Candida catenulata Candida intermedia Candida parapsilosis Candida phangngensis Candida silvae Candida tropicalis Debaryomyces hansenii Geotrichum candidum Kluyveromyces lactis Kluyveromyces marxianus Kodamaea ohmeri Rhodotorula mucilaginosa Saccharomyces cerevisiae Torulaspora delbrueckii Trichosporon* spp*. Trichosporon montevideense*	Brazil	Cardoso et al., 2015

### Traditional White-Brined Cheeses From Greece

One of the oldest and most popular types of white-brined cheeses is Feta, a traditional Greek cheese, classified as Protected Designation of Origin (PDO) (European Commission, 2002). Feta is a soft white cheese ripened in brine, with a rancid and slightly acid flavor, and a firm and smooth texture (Litopoulou-Tzanetaki and Tzanetakis, 2011). Traditionally, it is manufactured from non-pasteurized ovine milk or a mixture of ovine and caprine milk (up to 30%) (Table 1). At present, Feta is commercially made with pasteurized milk, using starter cultures *Streptococcus thermophilus* and *Lactobacillus delbrueckii* subsp. *bulgaricus* (Manolopoulou et al., 2003; Rantsiou et al., 2008). After coagulation and drainage, the curd is dry-salted for 4 – 5 days and, then, laid into the brine (8% NaCl) for ripening. After dry-salting, cheese remains on the cheese-table for approximately 15 days (warm ripening at 16–18°C). The rest of ripening period lasts for at least 60 days in the barrels at a cold storage temperature (Rantsiou et al., 2008; Litopoulou-Tzanetaki and Tzanetakis, 2011). In the early 1990s, yeast species of *Tetrapisispora blattae* (former name *Kluyveromyces blattae*), *Candida sphaerica* (anamorph of *K. lactis*), and *Lachancea thermotolerans* (former name *Kluyveromyces thermotolerans*) were isolated from brine of Feta cheese (Vivier et al., 1994). Manolopoulou et al. (2003) analyzed Feta cheese from three dairies in the Peloponnese Region (Southern Greece), showing that the total yeast counts in curd varied between the dairies (10^2^–10^3^ CFU/g) and generally increased at the dry room ripening period (2.6 × 10^3^–4.6 × 10^4^ CFU/g). Rantsiou et al. (2008) used culture-dependent and culture-independent techniques to characterize the microbiota of Feta cheeses produced by four different manufactures in Greece. The yeast counts at the most production sites were of 10^3^ CFU/g, presented by the dominant yeast species of *K. lactis* (79–83% of the total yeast population). The less abundant species were *P. fermentans*, *Pichia membranifaciens*, and *Candida zeylanoides*, occasionally isolated from single producers (16–20% of the total yeast population). The authors concluded that reduced diversity of yeast species in Feta was due to the adaptation to the particular environment of brine. According to Litopoulou-Tzanetaki and Tzanetakis (2011), yeasts and halotolerant microbes are the predominant microorganisms in fresh Feta cheese produced with non-pasteurized ewe’s milk. The total yeast counts, primarily dominated by *S. cerevisiae* and *D. hansenii*, were highest after the fourth day of ripening (7.2 × 10^5^ CFU/g) and, afterward, decreased during the ripening period (to 5.9 × 10^4^ CFU/g after 60 days of ripening).

Halloumi is a traditional white-brined cheese, produced mainly in Cyprus and Greece as a PDO product in accordance with the EU Regulation No 1151/2012 (European Commission, 2015) (Table 1). It is commonly made from pasteurized ovine or caprine milk (or a mixture of them), though cow milk can be added as well (Kamilari et al., 2020). Cheese blocks can be wrapped in dry leaves of *Mentha viridis*, giving the characteristic minty flavor to the cheese. Contrast to Feta, Halloumi’s blocks are heated after curd formation in whey at 90–95°C for at least 30 min. This step ensures the prevention of contamination, and it is essential for the characteristic flavor and elastic texture of Halloumi cheese (Kaminarides et al., 2009). There are two kinds of Halloumi cheese, the fresh and the mature (Kaminarides et al., 2009; European Commission, 2015; Kamilari et al., 2020). The latter type is immersed into the brine (14–16% w/v NaCl) for at least 40 days at 25°C before storage (Kaminarides et al., 2009; Kamleh et al., 2012). Yeasts *Candida* spp., *P. membranifaciens*, and *D. hansenii* were the most common contaminants, isolated from Halloumi cheese and brine in high counts (up to10^5^ CFU/g) (Mehyar et al., 2018).

Teleme is another traditional Greek cheese made with non-pasteurized milk, which is currently produced on an industrial scale, using yogurt commercial starters (Pappa et al., 2006). In Teleme processing, the curd is subjected to pressure to release whey and placed into the brine (18% w/v NaCl) for 20 h (Litopoulou-Tzanetaki and Tzanetakis, 2011). Laslo and György (2018) reported that the total counts of yeasts in Teleme comprised up to 8 × 10^3^ CFU/g. To our knowledge, there are no published studies on yeast identification in Teleme cheese.

### White-Brined Cheeses From Other European Countries

In many European countries, white-brined cheeses are produced from pasteurized cow’s milk and salted in brine without the use of dry-salting. Westall and Filtenborg (1998) isolated and identified spoilage yeasts in Feta-type cheese at three different dairies (A, B, and C) in Denmark (Table 1), showing high variation between the production sites. Predominant yeasts from the dairy A were *Torulaspora delbrueckii*, *G. candidum*, and *Y. lipolytica* (10^2^–10^6^ CFU/g), whereas *Candida boidinii*, *C. intermedia*, *C. zeylanoides*, *D. hansenii*, *K. lactis*, *K. marxianus*, *Pichia guilliermondii* (current name *Meyerozyma guilliermondii*), *P. membranifaciens*, and *Naumovozyma dairenesis* (formerly *Saccharomyces dairenesis*) were found in low counts (less than 10^2^ CFU/g). Yeast species isolated from the dairy B, belonged to *D. hansenii*, *Y. lipolytica*, and *Candida sake* (10^2^–10^6^ CFU/g), while *T. delbrueckii*, *C. zeylanoides*, *G. candidum*, *K. lactis*, and *P. guilliermondii* were sporadically found. Less diversity was observed among the yeast species from the dairy C, most of them belonging to *Candida aaseri* (formerly *Candida butyri*) and *Y. lipolytica* (10^3^–10^5^ CFU/g) (Westall and Filtenborg, 1998). Šuranská et al. (2016) characterized diversity and composition of yeast consortium in various Serbian artisanal white-brined cheeses (Table 1) with the use of traditional culturing and molecular techniques (ITS-RFLP, 26S rRNA amplicon sequencing and ITS–clone library restriction analysis). The total yeast counts in cheese increased during the ripening period of 10 days (from 10^4^ to 10^6^ CFU/g). Yeasts *D. hansenii*, *C. zeylanoides*, and *K. lactis* were isolated in high numbers (up to 10^6^ CFU/g) from most of the samples, followed by less abundant *T. delbrueckii*, *Trichosporon ovoides*, *Candida pararugosa*, *Y. lipolytica* and *G. geotrichum* (up to 10^3^ CFU/g). Additionally, yeast species not associated with the cheese making, e.g., *Cryptococcus albidus*, *Hanseniaspora uvarum*, and *Filobasidium globisporum*, were rarely found in a few samples. Another study with Serbian white-brined cheeses identified 17 yeast species, among them, *D. hansenii*, *C. zeylanoides*, and *T. delbrueckii* were predominant (Golić et al., 2013). The authors pointed out that the presence of rare species, such as *Trichosporon gracile*, *T. ovoides*, and *Torulaspora quercuum*, was due to the bad hygienic conditions during cheese production. Chipilev et al. (2016) analyzed white-brined cheeses from Bulgarian dairies produced with pasteurized cow milk and vacuum packaged after 45 days ripening period. More than 63% of cheeses were contaminated with yeasts, mostly *Candida* spp., *Rhodotorula* spp., and *Saccharomyces* spp., in total counts of 10^2^–10^6^ CFU/g.

### White-Brined Cheeses From Outside Europe

Serro Minas cheese is a traditional semi-hard cheese with acidic flavor, produced in Brazil from non-pasteurized bovine milk (Table 1). Whey of an older cheese covered with salt (known as “pingo”) is used as a natural starter (back slopping) in cheese-making (Cardoso et al., 2015). Serro Minas is ripened for 3 days at room temperature and, then, for 60 days at 10°C (Ministério da Agricultura Pecuária e Abastecimento (MAPA), 2000). Cardoso et al. (2015) reported higher counts and diversity of yeast species in Serro Minas cheese during the rainy season compared to the dry season (2.6 × 10^7^ CFU/g vs. 6.6 × 10^6^ CFU/g, respectively, after 15 days of ripening), indicating that the climate conditions and seasonal variation had an impact on the cheese microbiota. *D. hansenii* and *Kodamaea ohmeri* (formerly *Pichia ohmeri*) were predominant in Serro Minas cheese (1.3 × 10^8^ CFU/g and 7.9 × 10^6^ CFU/g, respectively, after 15 days of ripening in the rainy season). Other species, such as *Trichosporon montevideense*, *S. cerevisiae*, *G. candidum*, and *Rhodotorula mucilaginosa*, were referred as temporary contaminants, occasionally occurred in high numbers (up to 10^5^ CFU/g) throughout the ripening period. Furthermore, *C. parapsilosis*, *Candida tropicalis*, *G. candidum*, and *R. mucilaginosa* were only detected in the rainy season, while *Candida atlantica*, *C. intermedia*, *Candida phangngensis*, and *Candida silvae* were identified during the dry season (Cardoso et al., 2015).

In Africa, white-brined cheeses are mainly produced in the northern part. The most popular white-brined cheese in Egypt is Domiati, manufactured from buffalo or cow milk or their mixture (Table 1). Unlike the Greek Feta cheese, NaCl is added into the milk before coagulation and fermentation (Egyptian Organization for Standards and Quality (EOS), 2005; Ayad, 2009). According to Sharaf et al. (2014), 10 samples of Domiati cheeses out of 45 samples collected from the markets in Cairo, were contaminated with yeasts (up to 6 × 10^3^ CFU/g). Even higher yeast contamination levels (66% of samples, average counts of 2.63 × 10^5^ CFU/g) were recorded for Domiati in a later study (Hameed, 2016). The main yeast species isolated from Domiati were classified as *Candida albicans*, *Candida krusei*, and *D. hansenii*. Among them, *C. albicans* (detection frequency in Domiati cheese of 24%) is recognized as a human pathogen, commonly associated with poor sanitation and handling in cheese making (Hameed, 2016).

Mihalic cheese and the Turkish White cheese (Beyaz peynir) are the most consumed white-brined cheeses in Turkey, in which spoilage yeast consortia have been characterized (Table 1). Mihalic cheese is a semi-hard salty cheese with a pale creamy color and eye formation on the interior surface (Solak and Akin, 2013; Aday and Karagul Yuceer, 2014). It is traditionally manufactured from raw ovine, caprine, or cow milk without addition of starter cultures and ripened in wooden barrels filled with brine (16-18% v/v NaCl) for three months at 15-25°C (Solak and Akin, 2013; Karasu-Yalcin et al., 2017). Karasu-Yalcin et al. (2017) classified 72 yeast isolates from Mihalic cheese (29 samples in total), mainly belonging to the genera *Candida*, *Geotrichum*, and *Trichosporon*. The predominant species were halophilic *D. hansenii* (42% of the total isolates), along with *Candida cylindracea*, *C. inconspicua*, *Candida paludigena*, and *C. tropicalis*, found in lower numbers. In addition, *C. krusei*, *C. zeylanoides*, *G. candidum*, *Candida bertae*, *Candida catenulata*, *K. ohmeri*, *S. cerevisiae*, and *Trichosporon asahii* were rarely identified (Karasu-Yalcin et al., 2017). According to the recent report by Togay et al. (2020), the total yeast counts in Mihalic cheese of 4.1 × 10^3^–2.3 × 10^4^ CFU/g were dominated by *D. hansenii* and *Clavispora lusitaniae*. Production of Beyaz Peynir comprises 60–80% of the total cheese production in Turkey (Turkish Standards, 1995). It is a semi-hard cheese with acidic and/or salty flavor, produced from ovine, caprine, cow milk or mixture of them. The cheese blocks are commonly ripened in brine (14–16% NaCl) for 30–60 days at 12–15°C (Turkish Standards, 1995; Atasever et al., 2002; Hayaloglu et al., 2002, 2008). Yeasts species belonging to *Candida* spp., *K. lactis*, *Starmera amethionina* (formerly *Pichia amethionina* var. *amethionia*), *D. hansenii*, *C. zeylanoides*, and *T. delbrueckii* have been isolated from the Turkish White cheese in total counts of 2.45 × 10^3^ CFU/g (Öztürk and Şahin, 2000; Togay et al., 2020).

Akawi cheese belongs to the white-brined cheeses produced traditionally in Lebanon and Syria from pasteurized bovine and/or ovine milk (Hayaloglu et al., 2008; Ayyash et al., 2012; Hayaloglu, 2017). The curd is pressed in order to release the whey (Toufeili and Özer, 2006). High salt concentration in brine (20% w/w NaCl) and in the final product (9% w/w NaCl) ensures long shelf life of Akawi cheese (Pachlová et al., 2016). Halotolerant yeast strains of *D. hansenii* and *Candida guilliermondii* (anamorph form of *M. guilliermondii*, formerly *P. guilliermondii*) have been isolated from Akawi cheese produced traditionally in the Czech Republic (Pachlová et al., 2016). Khalil et al. (2018) isolated and identified yeast species from a traditional soft white-brined cheese Jiben-Al-Arab, manufactured from sheep milk in Mosul, Iraq. The cheese is typically stored in tins with brined whey at room temperature. The authors reported that most of the cheese samples were contaminated with low levels (<10^2^ CFU/g) of *Candida* spp. (*C. parapsilosis*, *C. albicans*, *C. tropicalis*, and *C. krusei*), *G. candidum*, and *Rhodotorula* spp. (Khalil et al., 2018).

Spoilage yeasts have been detected and enumerated in other types of white-brined cheeses without taxonomic identification. Among them, cheese Ezine has a Geograpical Indication status and registered as a unique trademark in Turkey (Türk Patent Enstitüsü, 2006; Uymaz et al., 2019). A seasonal (March – July) mixtures of pasteurized ewe milk (approximately 45-55%), goat milk (at least 40%), and cow milk (the most 15%) are used for the cheese making with addition of aromatic herbs (oregano, mint, thyme, etc.), which contribute to the characteristic flavor (Hayaloglu et al., 2008). Ezine is produced without addition of starter cultures and ripened for a long period (8–12 months). Fungal average counts of 3.2 × 10^3^ CFU/g have been reported during ripening of Ezine cheese from three different Turkish regions (Uymaz et al., 2019). Another example is a traditional white-brined cheese Nabulsi, popular in Jordan and the Middle East. During production, the rennet is added into non-pasteurized milk (cow, sheep, goat milk, or their mixture) without the use of starter culture, and the curd is allowed to set for 35–40 min before cutting. Then, the curd is boiled in the brine solution (18–20% w/v NaCl), filled in cans with brine, and stored at room temperature for up to 2 years (Al-Dabbas et al., 2014; Sabbah et al., 2019). Yeast counts of 3.6 × 10^5^ CFU/g in Nabulsi cheese have been reported (Al-Dabbas et al., 2014).

## Properties and Spoilage Potential of Yeasts

### Proliferation of Spoilage Yeasts

Proliferation of spoilage yeasts in dairy products depends on a range of extrinsic factors, as temperature and humidity, particularly during storage, as well as intrinsic factors, such as milk composition, water activity, NaCl content, pH, and antimicrobials (Carrasco et al., 2006). In addition, the co-existing starter cultures and commensal microorganisms might influence the growth of spoilage yeasts. Thus, glucose and galactose, generated from lactose degradation by LAB, can be subsequently utilized by yeasts, otherwise incapable to ferment lactose, and promote their growth (Álvarez-Martín et al., 2008). In white-brined cheeses, yeasts may have detrimental effects on the final products by releasing off-flavors (bitterness, fruity, rancid, soapy), producing gas (CO_2_), or causing discoloration and textural changes (Soliman and Aly, 2011; Padilla et al., 2014; Gonçalves Dos Santos et al., 2017; Al-Gamal et al., 2019). It should be noted, however, that yeasts in low numbers are rarely a problem, since the counts need to exceed 10^4^-10^6^ CFU/g, before the quality defects can be detectable (Laslo and György, 2018; Tokak et al., 2019).

Miloradovic et al. (2018) investigated the effect of packaging conditions on yeast growth in white-brined cheese produced in Serbia with goat milk. Before packaging, the cheese was ripened in two different salt concentrations (3% and 6% w/v NaCl) for 10 days, and, afterward, it was either vacuum packaged or kept in a modified atmosphere (MAP) (60% CO_2_, 40% N_2_) for 40 days at storage temperature. The brine strength had no effect on yeast counts, determined at the end of the brining period (10th day). Concurrently, the MAP was more effective for reduction of yeast numbers at the end of storage, resulting in lower yeast counts of 10^3^–10^4^ CFU/g, compared to 10^5^ CFU/g in the vacuumed samples.

Using mathematical models, Vivier et al. (1994) described the species variation and the impact of pH, temperature, NaCl concentration, and water activity on the growth of *T. blattae*, *C. sphaerica*, and *L. thermotolerans*, isolated from brine of Feta cheese. All tested yeast species were able to grow at salt content up to 16%, low a_w_ of 0.94 and in the temperature range of 4–37°C. Among the yeast species, *C. sphaerica* was the most salt-tolerant (grown at 16% NaCl), while *L. thermotolerans* was the most resistant to high temperatures (grown at 41°C). In another comparative study with dairy yeasts, Praphailong and Fleet (1997) demonstrated that *D. hansenii* and *Pichia anomala* were the most halotolerant, able to grow at 15% NaCl, whereas growth of *K. marxianus* and *S. cerevisiae* was inhibited at 7.5% and 10% NaCl, respectively. It was further reported that pH variations within a range of pH 3.0 to 7.0 had insignificant impact on the growth of dairy-related yeasts *C. sphaerica*, *D. hansenii*, *Y. lipolytica*, *K. marxianus*, *L. thermotolerans*, *P. membranifaciens*, *P. anomala*, *S. cerevisiae*, and *T. blattae* (Vivier et al., 1994; Praphailong and Fleet, 1997).

### Proteolytic and Lipolytic Activity

**FIGURE 1:**
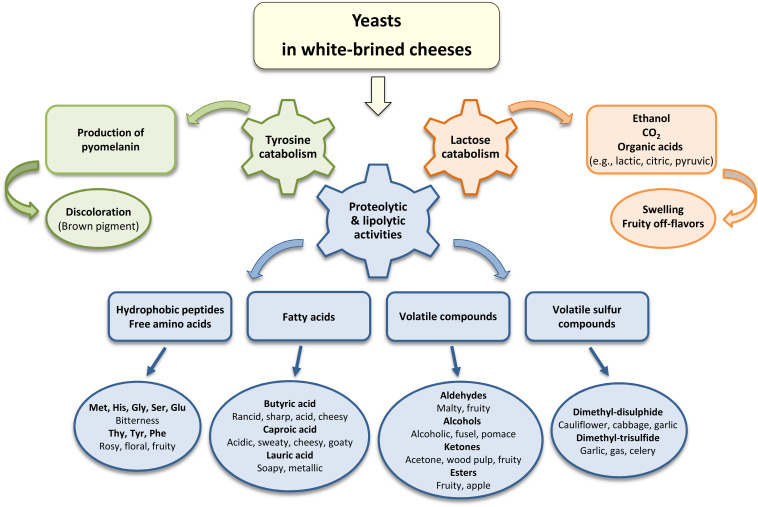
Association between the metabolites produced by yeasts and the quality defects in white-brined cheeses, i.e., off-flavors, discoloration and swelling.

Yeast’s proteolytic and lipolytic activities (Figure 1) are the main factors, influencing the organoleptic characteristics and causing deterioration of white-brined cheeses (Sørensen et al., 2011; Karasu-Yalcin et al., 2017; Tokak et al., 2019). Yeasts have the ability to convert milk proteins and fat into amino acids and free fatty acids (FFA), the precursors of aroma and flavor compounds (Chen et al., 2010). In particular, the short-chain fatty acids (butyric and caproic) and the middle chain fatty acids (lauric and myristic) contribute to sour, rancid, sharp, and soapy flavor in cheese (Aday and Karagul Yuceer, 2014; Tokak et al., 2019). In addition to FFA, activity of yeast lipolytic enzymes (lipases, esterases, etc.) leads to release of the volatile aroma compounds, e.g., alcohols, aldehydes, ketones, and esters (Karasu-Yalcin et al., 2017). Formation of these compounds is associated with fruity, fusel, wood pulp, pomace, and butterscotch flavors (Sørensen et al., 2011). Sørensen et al. (2011) estimated production of volatile compounds by *Y. lipolytica* CBS 2075, *D. hansenii* D18335 and *S. cerevisiae* D7 grown in cheese medium at variable temperatures (12°C and 25°C) and NaCl concentration (0% and 3% w/v). The short-chain methyl-ketones (2-propanone, 2-butanone, 2-pentanone, and 3-methyl-2-pentanone) were primarily produced by *Y. lipolytica*. Concurrently, *D. hansenii* produced the highest levels of aldehydes (2-methylpropanal, 2-methylbutanal, and 3-methylbutanal) and alcohols (2-methyl-1-propanol and 2,3-methyl-1-butanol), while *S. cerevisiae* contributed to esters (ethyl-propionate and ethyl-butanoate). Interestingly, the authors observed that release of the volatile compounds by yeasts was highly influenced by the temperature and salt content in the growth medium (Sørensen et al., 2011).

Milk caseins are hydrolyzed through the synergistic action of acid phosphatase and proteolytic enzymes. High activity of acid phosphatase, converting phosphates to small peptides and free amino acids, contributes to flavor development in the acidic environment of matured cheeses (Karasu-Yalcin et al., 2017). Bitterness is the main flavor defect in cheese, commonly correlating with the proteolytic activity of yeasts and associated with the formation of low molecular weight hydrophobic peptides. Additionally, other metabolites, e.g., amino acids, amines, amides, long-chain ketones, and mono-glycerides, produced by *Candida* spp., *K. lactis*, *S. amethionina*, and *D. hansenii*, promote the bitter flavor in white-brined cheese (Öztürk and Şahin, 2000; Aday and Karagul Yuceer, 2014; Ganesan and Weimer, 2017). The cheese flavor is also affected by the volatile sulfur compounds, such as methanethiol and sulfides, generated from milk caseins through the amino acid metabolic pathways (methionine and cysteine) (Aday and Karagul Yuceer, 2014; Laslo and György, 2018). Sulfides (dimethyl disulfide, DMDS, and dimethyl trisulfide, DMTS), released via metabolic activity of *Y. lipolytica*, correlated with cabbage and garlic flavors in cheese medium (Sørensen et al., 2011). The ability to produce different types of sulfides has been demonstrated for strains of *G. candidum*, *Y. lipolytica*, *D. hansenii*, and *S. cerevisiae* grown in a model cheese medium or laboratory medium, supplemented with amino acids (methionine and cysteine) (Arfi et al., 2002, 2004; López Del Castillo-Lozano et al., 2007).

Lipolytic and proteolytic properties have been characterized for several potential yeast spoilers and commonly reported as strain-dependent. Among the 199 yeasts from Serro Minas cheese (Brazil), only a minor part of isolates of *K. marxianus*, *D. hansenii*, and *K. ohmeri* (5–10%) displayed lipase and protease activity. Likewise, isolates of *G. geotrichum* and *R. mucilaginosa* showed either lipolytic or proteolytic activity, respectively (Cardoso et al., 2015). Extracellular lipase activity of *Y. lipolytica* was determined in Serbian white-brined cheeses by Golić et al. (2013). According to Westall and Filtenborg (1998), the lipolytic activity of *Y. lipolytica* and other yeast species found in high numbers (10^5^ CFU/g), accounted for texture softening of the Danish Feta-type cheese. Among the isolates of *Candida* spp. from the white-brined cheese Jiben-Al-Arab (Iraq), *C. parapsilosis* and *C. tropicalis* were distinguished from *C. albicans* and *C. krusei* by high phospholipase activity, while *C. tropicalis* was the only species exhibiting esterase activity (Khalil et al., 2018). Karasu-Yalcin et al. (2017) reported that most of the yeast isolates (30 in total) from Mihalic cheese, belonging to *G. candidum*, *T. asahii*, and *Candida* spp., had esterase activity, while lipase activity was only detected for a few strains of *C. tropicalis*, *D. hansenii*, and *G. candidum*.

### Lactose metabolism

Production of CO_2_ and ethanol, the major by-products of yeast metabolism (Figure 1), might result in swelling of cheese cans (Bintsis et al., 2000). Besides, fruity flavor may develop when ethanol reacts with short-chain fatty acids to form a variety of esters (Laslo and György, 2018). Gas formation has been detected in cheese containers or packages, when lactose-fermenting yeasts reached typically more than 10^6^ CFU/g (Bintsis et al., 2000; Öztürk and Şahin, 2000; Fadda et al., 2001; Sharaf et al., 2014). Several studies identified spoilage yeasts in white-brined cheeses in connection with the incidents of swollen packages. For instance, *Candida* spp., *K. lactis*, *S. amethionina*, and *D. hansenii*, determined in Turkish white-brined cheese, were capable to cause gas formation in the products even at high amounts of NaCl (12 % w/w) (Öztürk and Şahin, 2000). Fadda et al. (2001) reported that *Dekkera anomala* (a strongly fermenting yeast) and *K. lactis* were responsible for swelling of Sardinian Feta cheeses. Earlier industrial survey on Feta cheese demonstrated that yeast species of *T. blattae*, *C. sphaerica*, and *L. thermotolerans*, isolated from brine, caused gas formation in the packaged products (Vivier et al., 1994). Swelling of the packages of Feta-type cheese during storage at the Danish dairies was ascribed to lactate utilization and propagation of *T. delbrueckii* (Westall and Filtenborg, 1998).

### Discoloration and Tyrosine Metabolism

**FIGURE 2:**
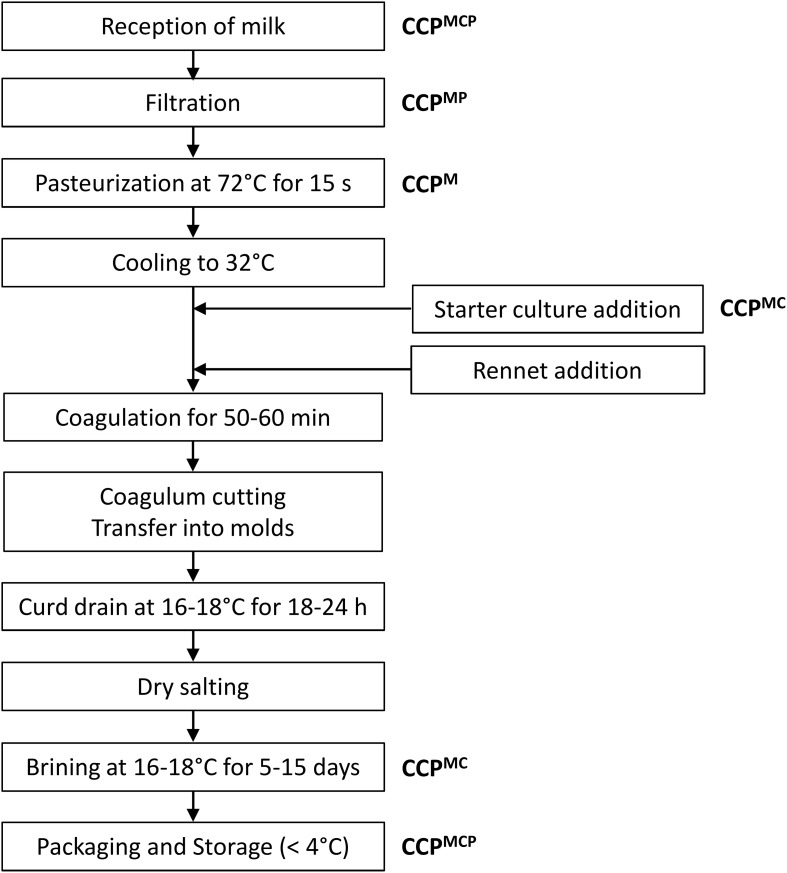
Flow-chart of Feta cheese production with indicated Critical Control Points (CCP) of the microbiological^M^, chemical^C^ and physical^P^ hazards [modified from Mauropoulos and Arvanitoyannis (1999); Tamime et al. (2007) and El-Hofi et al. (2010)].

Formation of brown pigments on cheese surfaces is commonly attributed to *Y. lipolytica* (Figure 1). To our knowledge, at least one publication reported discoloration of the white-brined cheese Domiati (Sharaf et al., 2014), though similar defects have been observed in other types of cheeses, e.g., soft surface-ripened cheeses, Mozzarella, Ricotta, and Gouda (Casalinuovo et al., 2015; Chierici et al., 2016; Igoshi et al., 2017). Cheese presents a suitable substrate for pigment production, as it contains lactic acid, amino acids (e.g., glycine, asparagine, glutamine), glucose, and manganese, promoting the browning effect. On the opposite, the increased content of glucose correlated with a delay of browning in tyrosine-containing laboratory medium (Carreira et al., 2001a,b). The brown pigments, known as melanins, are produced by yeasts from L-tyrosine via dihydroxyphenylalanine (DOPA) pathway or through accumulation and autoxidation of a pigment precursor, homogentisic acid (HGA) (Carreira et al., 2001b; Schmaler-Ripcke et al., 2009; Ben Tahar et al., 2020). In *Y. lipolytica*, production of pigments includes synthesis and accumulation of HGA, followed by convertion of HGA into benzoquinone acetic acid by autoxidation, which is further turned into pyomelanin through self-polymerization (Figure 2) (Ben Tahar et al., 2020). Recently, Ben Tahar et al. (2020) verified production of melanin pigments in *Y. lipolytica* W29 via L-tyrosine catabolism. Interestingly, these pigments exhibited both antioxidant and antimicrobial activities, indicating perspectives for biotechnological applications of *Y. lipolytica* (Ben Tahar et al., 2020).

### Production of Biogenic Amines

Biogenic amines (BA) are toxic metabolites, commonly arising in fermented foods from decarboxylation of free amino acids due to microbial activity. The most abundant BA in cheese are histamine, tyramine, cadaverine, putrescine, tryptamine, and phenylethylamine, produced mainly by lactic acid bacteria, *Enterobacteriaceae*, and, to a lesser extent, by *D. hansenii*, *Y. lipolytica*, *K. marxianus*, *S. cerevisiae*, and *Candida* spp. (Benkerroum, 2016). BA are indicators of cheese quality and safety, since, in significant amounts, they can cause adverse effects on human health, e.g., fluctuation of blood pressure, headache, vomiting, and diarrhea (Ruiz-Capillas and Herrero, 2019). According to the European Food Safety Authority (EFSA), consumption of 50 mg histamine and 600 mg tyramine (per person per meal) are considered safe for healthy individuals (EFSA, 2011). The United States Food and Drug Administration (FDA) set the maximum levels of histamine to 50 mg/kg in fishery products (FDA, 2020). Safety criteria for other BA are not covered by specific legislation.

Biogenic amines have been found in Feta (Valsamaki et al., 2000), Akawi cheese (Pachlová et al., 2016), Beyaz peynir (Durlu-Özkaya et al., 1999), Iranian white brined cheese (Aliakbarlu et al., 2011; Rohani et al., 2013), and other varieties of white-brined cheeses and ripened cheeses collected from the European dairies and small-scale farms (Bunkova et al., 2013; Combarros-Fuertes et al., 2016; Bonczar et al., 2018; Espinosa-Pesqueira et al., 2018; Mayer and Fiechter, 2018). Based on diverse research, EFSA published the mean values of histamine (21–62 mg/kg), tyramine (68–104 mg/kg), putrescine (25–65 mg/kg), and cadaverine (72–109 mg/kg) recorded in cheese (EFSA, 2011). Pachlová et al. (2016) reported that the total BA (putrescine, histamine, tyramine and cadaverine) in several batches of traditionally manufactured cheese Akawi (Czech Republic) exceeded 120 mg/kg. Yeast strains of *D. hansenii* and *C. guilliermondii*, isolated from Akawi, were tested positive for decarboxylase activity, indicating that they might contribute to BA accumulation during the long storage periods. The authors further pointed out that decarboxylase activity in yeasts was strain-dependent and, particularly, putrescine and cadaverine were predominant BA produced in high levels (50–1000 mg/L) in laboratory medium (Pachlová et al., 2016). Strain specificity in BA production by microorganisms is well recognized (Benkerroum, 2016). Bäumlisberger et al. (2015) demonstrated that certain strains of *D. hansenii* and *Y. lipolytica* had an ability to metabolize a broad spectrum of BA due to a peroxisomal amine oxidase activity, and suggested that BA non-producer strains had a potential to reduce BA in fermented foods (Bäumlisberger et al., 2015).

In cheese, production of BA is highly influenced by amino acid availability, the presence of contaminating microorganisms, as well as processing and storage conditions. Occurrence of BA can be reduced or prevented by proper hygiene practices, milk pateurization, low pH, optimized ripening conditions (e.g., high salt content), controlled packaging (e.g., vacuum pakaging), and low storage temperatures (Valsamaki et al., 2000; Novella-Rodríguez et al., 2002; Aliakbarlu et al., 2011; Andiç et al., 2011; Bunkova et al., 2013; Gardini et al., 2016). Aliakbarlu et al. (2011) performed modeling of the BA content in Iranian white brined cheeses as a function of processing variables, such as ripening time (25–75 days), temperature (4–14°C), and brine concentration (10–13%). At low level of ripening time, the BA content decreased with increasing levels of brine concentration, while at high level of ripening time, brine concentration had inverse effect, which was explained by the softening of the texture and diffusion of BA to brine (Aliakbarlu et al., 2011).

## Bio-Protective Cultures and Other Yeast Interactions

### Bio-Protective Cultures

The dairy market alongside with consumer’s demands continues to move towards more natural products, i.e., less processed and free from artificial ingredients (“clean label”) (Román et al., 2017). Finding clean-label solutions for the preservation of white-brined cheeses is particularly important, regarding their susceptibility to yeast spoilage. In this connection, bio-protective cultures offer a promising alternative to chemical preservatives (Crowley et al., 2013). Bio-protective cultures are referred as food cultures, deliberately applied as live microorganisms to control the microbiological status in food (EFFCA, 2018; Ben Said et al., 2019). As food additives, bio-protective cultures should have “generally recognized as safe” (GRAS) status and be included in the qualified presumption of safety (QPS) list in Europe (Ben Said et al., 2019). In addition to yeast inhibition, bio-protective cultures should be able to proliferate in dairy products without affecting the performance of other starter and adjunct cultures, and without changing the technological and sensory quality of the products (Schnürer and Magnusson, 2005). Lactic acid bacteria (LAB) associated with milk fermentation, specifically within the genus *Lactobacillus*, are well known for their preserving effects (Bernardeau et al., 2008). Various commercial protective cultures, consisting of selected strains of LAB, have been developed and applied in dairy production, among them, FRESHQ® (Chr. Hansen) and HOLDBAC® YM (DuPont) targeting yeasts and molds in cheeses and other fermented dairy products.

### Interactions Between Spoilage Yeasts and LAB

Inhibitory properties of LAB against potential spoilage yeasts have been examined for several fermented milk products, mainly yogurts, sour cream, and surface-ripened cheese (Corsetti et al., 2001; Crowley et al., 2013; Delavenne et al., 2013; Laèanin et al., 2017; Fröhlich-Wyder et al., 2019; Garnier et al., 2019). The studies commonly pointed out that the antifungal activity of LAB was strain-specific and depended on the target yeast species and/or strains. Table 2 presents examples of the LAB strains, mixed cultures and yeasts, producing antifungal metabolites and inhibiting potential yeast spoilers in white-brined cheeses. In the following, new taxonomic names of LAB (shown in brackets) are referred in accordance with Zheng et al. (2020). Leyva Salas et al. (2018) performed a large-scale screening assay of antifungal activity of LAB in yogurt and cheese matrix, among them, *Lactobacillus harbinensis* (*Schleiferilactobacillu*s *harbinensis*), *Lactobacillus plantarum* (*Lactiplantibacillus plantarum*), *Leuconostoc mesenteriodes*, *Lactococcus lactis*, *Lactobacillus rhamnosus* (*Lacticaseibacillus rhamnosus*), and *Propionibacterium jensenii* (*Acidipropionibacterium jensenii*), against potential spoilage yeasts (*G. geotrichum* and *Y. lipolytica*). The authors developed two antifungal combinations of mixed cultures, consisting of *L. plantarum* L244, *S. harbinensis* L172 and/or *L. rhamnosus* CIRM-BIA1113, suitable for application as adjunct cultures in sour cream and semi-hard cheese. According to Delavenne et al. (2013), *S. harbinensis* had the strongest antifungal effect in milk and yogurt, compared to *Lactbacillus casei* (*Lacticaseibacillus casei*), *Lactobacillus paracasei* (*Lacticaseibacillus paracasei*), and *L. rhamnosus*, completely inhibiting all tested yeast strains of *D. hansenii*, *K. lactis*, *K. marxianus*, *R. mucilaginosa*, and *Y. lipolytica*.

**TABLE 2:** Inhibitory activity of lactic acid bacteria, yeasts and their metabolites against yeast species, frequently occurring as contaminants in white-brined cheeses.

Microorganisms	Dairy matrix/Media	Targets	Metabolites	References
**Lactic acid bacteria (LAB)**				
*Schleiferilactobacillus harbinensis* K.V9.3.1 Np	Milk, yogurt, and fermented milk	*Debaryomyces hanseii Kluyveromyces lactis Kluyveromyces marxianus Rhodotorula mucilaginosa Yarrowia lipolytica*	Polyamides; organic acids (acetic, benzoic, lactic, 2-pyrrolidone-5-carboxylic, hexanoic, 2-hydroxybenzoic)	Delavenne et al., 2013, 2015; Mieszkin et al., 2017; Mosbah et al., 2018
*Acidipropionibacterium jensenii* CIRM-BIA1774	Sour cream and semi-hard cheese	*Galactomyces geotrichum Yarrowia lipolytica*	Propionic acid; Acetic acid	Garnier et al., 2019, 2020
*Lacticaseibacillus rhamnosus* CIRM-BIA1952	Sour cream and semi-hard cheese	*Galactomyces geotrichum Yarrowia lipolytica Rhodotorula mucilaginosa*	Lactic acid; Acetic acid Bioactive peptide (RLNFLKKIS)	Garnier et al., 2019, 2020
**Mixed cultures**				
*Lactiplantibacillus plantarum* L244 *Schleiferilactobacillus harbinensis* L172 *Lacticaseibacillus rhamnosus* CIRM-BIA1113	Sour cream and semi-hard cheese	*Galactomyces geotrichum Yarrowia lipolytica*	Not reported	Leyva Salas et al., 2018
*Lacticaseibacillus rhamnosus Lacticaseibacillus paracaseii*	Fermented milk	*Debaryomyces hanseii Torulaspora delbrueckii Rhodotorula mucilaginosa*	Not reported	Siedler et al., 2020
*L. plantarum* DSA 20174 *Lactobacillus helveticus* CNRZ 32	M17/MRS	*Candida parapsilosis*	Organic acids	Al-Gamal et al., 2019
*Lacticaseibacillus paracasei* CH127 *Lacticaseibacillus rhamnosus* CH126	Sour cream	*Debaryomyces hansenii*	Bioactive peptide (DMPIQAFLLY)	McNair et al., 2018
**Yeasts**				
*Yarrowia lipolytica* 1E07	Cheese model	*Geotrichum candidum* 3E17	Ammonium; Proline	Mounier et al., 2008
*Debaryomyces hansenii* 1L25	Liquid cheese medium	*Yarrowia lipolytica* 1E07	Not reported	Malek et al., 2018
*Debaryomyces hansenii*	YEPD-methylene blue agar, and commercial cheeses (Romano and blue cheese)	*Yarrowia lipolytica Wickerhamomyces anomalus Candida tropicalis Candida albicans*	Mycocins	Banjara et al., 2016; Al-Qaysi et al., 2017
*Kluyveromyces lactis*	YPD agar	*Saccharomyces cerevisiae*	Zymocin	Jablonowski et al., 2001; Lu et al., 2007; Lentini et al., 2018
*Lindnera saturnus*	Cheese	*Saccharomyces cerevisiae* VL1 *Kluyveromyces marxianus* ATCC8640	Not reported	Liu and Tsao, 2009

Major mechanisms behind the antimicrobial activities of LAB in dairy products include competition for the limited amount of nutrients (competitive exclusion), decrease of pH due to lactic acid production, and release of antifungal compounds, such as organic acids, fatty acids, peptides, and hydrogen peroxide (Álvarez-Martín et al., 2008; Siedler et al., 2019, 2020; Garnier et al., 2020). Recently, Siedler et al. (2020) demonstrated that competitive exclusion through depletion of the essential trace element manganese was the main factor, accounting for inhibition of *D. hansenii*, *T. delbrueckii*, and *R. mucilaginosa* by *L. rhamnosus* and *L. paracasei*. The authors established a link between the differences in expression of the manganese transporter (MntH1) and the protective effect of the tested LAB, and proposed that manganese scavenging might be a common trait within *Lactobacillus* spp. Al-Gamal et al. (2019) reported that inhibitory effect of *L. plantarum* DSA20174 and *Lactobacillus helveticus* CNRZ32 on the growth of *C. parapsilosis*, isolated from Egyptian Feta-type cheeses, was related to excessive production of organic acids by LAB. The recent mechanistic studies in fermented milk and yogurt demonstrated that *S. harbinensis* K.V9.3.1Np produces both polyamines and organic acids (acetic, lactic, 2-pyrrolidone-5-carboxylic, hexanoic, benzoic, and 2-hydroxybenzoic), leading to membrane disruption and cell lysis of *Y. lipolytica* (Delavenne et al., 2015; Mieszkin et al., 2017; Mosbah et al., 2018). McNair et al. (2018) isolated and characterized a bioactive peptide (DMPIQAFLLY) derived from β-casein in sour cream added bioprotective cultures (*L. paracasei* CH127 and *L. rhamnosus* CH126). The peptide targeted *D. hansenii*, attenuating its growth rate and affecting cell morphology (smaller and denser colonies). Of particular interest is an extensive research by Garnier and co-workers, who characterized antifungal activities of LAB (430 strains in total) against yeasts (*G. geotrichum* and *Y. lipolytica*), using cheese mimicking model, and identified a broad spectrum of potential antifungal compounds in selected dairy fermentates of *L. rhamnosus* CIRM-BIA1952 and *A. jensenii* CIRM-BIA1774 (Garnier et al., 2019, 2020). Overall, more than 50 compounds (organic acids, fatty acids, volatile compounds, and peptides), produced by specific LAB, have been identified. Among the major organic acids, lactic and acetic acids were most abundant in *L. rhamnosus* CIRM-BIA1952 fermentate, while propionic and acetic acids were associated with *A. jensenii*. Furthermore, α_s2_-casein-derived peptides (pepa4c177 or RLNFLKKIS) identified in *L. rhamnosus* CIRM-BIA1952 fermentate, possessed the ability to inhibit *R. mucilaginosa* (Garnier et al., 2020).

### Other Microbial Yeast Interactions

Studies on interactions within yeast consortium have been preferably focused on surface-ripened cheeses and fermented milk products, however, similar interaction mechanisms are foreseen in white-brined cheeses (Fröhlich-Wyder et al., 2019). Interactions within the yeast communities are facilitated by several communication mechanisms referred as quorum sensing (QS), which regulate such fungal behaviors as growth, sporulation, biofilm production, secretion of virulence factors, etc. (Mehmood et al., 2019). Aromatic alcohols generated via the amino acids metabolic pathways, e.g., tryptophol, tyrosol, and phenylethanol, are the most common QS molecules identified in yeasts, e.g., *S. cerevisiae* (Avbelj et al., 2015, 2016). Gori et al. (2007) reported that dairy yeasts *D. hansenii*, *S. cerevisiae* and *Y. lipolytica* produced ammonium compounds, which acted as signaling molecules, affecting development of the neighboring yeast colonies of the same species on cheese agar plates.

Negative interactions between *Y. lipolytica* and other dairy yeasts, such as *G. candidum* and *D. hansenii* in model cheeses have been reported (Mounier et al., 2008). The yeast *Y. lipolytica* inhibited hyphal formation and caused morphological changes (spaghetti-like structures) in *G. candidum*, possibly, due to the high amounts of ammonium and proline produced by *Y. lipolytica*, along with reduced metabolic efficiency in *G. candidum* (Mounier et al., 2008). Recently, Malek et al. (2018) used transcriptomic analysis to elucidate the interactions between *D. hansenii* co-cultured with *Y. lipolytica*. The study demonstrated that growth inhibition of *D. hansenii* was related to a decrease in mitochondrial respiratory chain functioning, and, consequently, to a programmed cell death, rather than ammonia production or competition for nutrients.

Certain dairy-related yeast species are capable to secrete toxins exhibiting antagonistic activities against other sensitive yeasts (killer toxins or mycocins). Mechanisms behind the action of killer toxins include disruption of membrane integrity, blocking of the DNA synthesis, and/or mRNA translation (Mannazzu et al., 2019). Currently, four killer toxins (K1, K2, K28, and Klus) have been identified in *S. cerevisiae* and differentiated by receptor sites, killing mechanisms, and lack of cross-immunity (Orentaite et al., 2016; Becker and Schmitt, 2017; Gier et al., 2020). The killing activity of the most well-studied toxin K1 is based on binding to the β-1,6-D-glucan on a target cell and disruption of cytoplasmic membrane by forming the cation-selective ion channels (Gier et al., 2020). Dairy isolates of *D. hansenii* produced mycocins, targeting *Y. lipolytica*, *Wickerhamomyces anomalus*, *C. tropicalis*, and *C. albicans* (Banjara et al., 2016; Al-Qaysi et al., 2017; Çorbacı and Uçar, 2017). Studies with *C. albicans* mutants defective in MAPK kinase pathways suggested that, specifically Hog1 phosphorylation site and the kinase activity, were implicated in the resistance of *C. albicans* to *D. hansenii* killer toxins (Morales-Menchén et al., 2018). Lethality of toxin zymocin, secreted by the dairy yeasts *K. lactis*, involves cleavage of tRNA in *S. cerevisiae* (Lu et al., 2007; Lentini et al., 2018). Liu and Tsao (2009) reported that yeast species *Lindnera saturnus* (former name *Williopsis saturnus)* reduced the growth of lactose and/or galactose fermenting spoilage yeasts *S. cerevisiae* VL1 and *K. marxianus* ATCC8640 in cheese, possibly, via competitive exclusion or production of killer toxins.

## Measures to Prevent Contamination and Proliferation of Spoilage Yeasts in White-Brined Cheeses

### Quality Assurance

**FIGURE 3:**
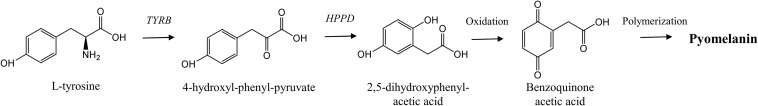
Metabolic pathways of pyomelanin production by *Yarrowia lipolytica*. Abbreviations: *TYRB*, tyrosine aminotransferase; *HPPD*, 4-hydroxyl-phenyl- pyruvate dioxygenase [modified from Schmaler-Ripcke et al. (2009), Ben Tahar et al. (2020)].

To assure the quality and safety of dairy products, several preventive and control strategies can be applied during processing, storage and handling of the finished product [reviewed by Garnier et al. (2017a)]. These strategies rely on the universal management systems, such as the Hazard Analysis Critical Control Points (HACCP) system, Good Manufacturing Practice (GMP), Good Hygienic Practice (GHP), and the International Organization for Standardization (ISO) 22000 (Papademas and Bintsis, 2010; Tamime et al., 2011; FACEnetwork, 2016; ISO, 2018; Awasti and Anand, 2020). Preventive strategies aim to avoid yeast cross-contamination during production, using such technologies as milk pasteurization, air filtration, sanitation, aseptic packaging conditions, etc. Control strategies are based on the inhibition of yeast growth by chemical preservation, the addition of protective cultures, refrigeration, or modified atmosphere packaging. Based on the principles of HACCP and GMP, dairies have developed site-specific regulations and sanitation procedures. Implementation of HACCP analyzes in the production of Feta-type cheeses have been reported (Mauropoulos and Arvanitoyannis, 1999; Tamime et al., 2007; El-Hofi et al., 2010; Carrascosa et al., 2016; Kapshakbayeva et al., 2019). For instance, Kapshakbayeva et al. (2019) performed a systematic analysis of hazards, their causes, consequences, and preventive measures throughout the production line of the white-brined cheese type Halloumi, and identified 10 risk factors and the Critical Control Points (CCP) in the individual production step (equipment, raw milk, pasteurization, enzyme application, curd processing, molding, salting, and packaging). Figure 3 shows the flow chart of Feta cheese production, in which the main procedures with potential critical hazards (microbiological, chemical and physical) are indicated as CCP [modified from Mauropoulos and Arvanitoyannis (1999)]. Microbiological hazards may be caused by the growth of psychrotrophic microorganisms and pathogens (raw milk), presence of bacteriophages (from starter cultures and/or environment), and cross-contamination with spoilage microorganisms after milk pasteurization. Chemical hazards may result from the presence of toxins, residues of detergents, disinfectants, and other chemical substances, while physical hazards are typically associated with penetration of extraneous material from the environment, e.g., parts of the equipment and packaging material (Tamime et al., 2007; El-Hofi et al., 2010).

In the case of documented yeast-spoilage problems in the dairy industry, a root cause analysis (RCA) need to be conducted as a part of the industrial process control (BRC Global Standards, 2012). The purpose of RCA is to trace the origin(s) of the yeast contamination in order to implement appropriate preventive measures to address the spoilage problem and food safety in general. The next step is to employ long-term corrective actions to address the root cause, identified during RCA, and to ensure that the problem does not recur. In practical circumstances, however, there may be more than one root cause for a given spoilage incident (e.g., related to faulty equipment, improper processing, personnel faults, improper ingredients or environmental issues), and this multiplicity can hamper the establishment of the causal graph.

### Routes of Contamination

Depending on the design of production plants and in case of open facilities, yeast contamination of heat-treated milk can occur if sub-optimal air quality conditions prevail in the production environment. For instance, due to the pressure differences, contaminated air from the surrounding environment can enter product vessels via the incubation tanks. In practice, two principal approaches can minimize this risk: (a) optimally, the entire processing environment should be rendered a “clean area” via the use of High-Efficiency Particulate Air (HEPA) filters. In terms of clean-room classification, these HEPA filters should be of at least ISO 8 class number, according to ISO 14644-1; (b) in case the former is not a feasible option due to constructive restrains or financial limitations, the establishment of ‘local’ clean areas around exposed equipment (i.e., the establishment of “clean” mini-environments that protect equipment such as filling and packaging equipment) should be implemented (ISO, 2015).

Pasteurization of milk will, in principle, destroy all yeast cells, including yeast spores, as they are generally not heat resistant. However, in some countries, e.g., Greece, good quality raw milk can be used for the manufacture of Feta cheese, provided that the cheese undergoes proper ripening for at least two months. However, to our knowledge, raw-milk Greek Feta cheese manufacture is only practiced by small artisanal cheesemakers. In contrast, large dairy companies pasteurize the milk before cheese making (75°C for 15 s), which effectively controls microbial food safety hazards (Tamime et al., 2007). Hence, besides the inactivation of pathogens, the heat-treated milk should be essentially devoid of viable vegetative cells (bacterial and fungal). Consequently, with respect to white-brined cheeses, the problems with yeast spoilage mainly originate from environmental contamination(s) at some production stage(s) after the heat-treatment step. This has been stated by several studies in dairy plants, which pointed out that production facilities and, specifically, the contaminated air are major sources of yeast contamination of white-brined cheeses (Temelli et al., 2006; Beletsiotis et al., 2011; Stobnicka-Kupiec et al., 2019; Kandasamy et al., 2020). Stobnicka-Kupiec et al. (2019) identified *C. albicans*, *Candida glabrata*, *C. rugosa*, *D. hansenii*, *G. candidum*, *Rhodotorula* spp., and *Y. lipolytica* from the air and surface samples of commercial and traditional Polish dairy plants. The highest concentration of yeast and molds was found in worktops in milk reception, cheese production area, and in the air samples (50–480 CFU/m^3^). Another one-year prospective study in a Greek dairy plant showed that an average fungal load, presented by *Cladosporium* spp., *Penicillium* spp., and unidentified yeasts, comprised 362.3 CFU/m^3^ in outdoor air and up to 266.2 CFU/m^3^ in the indoor locations (Beletsiotis et al., 2011). Substantial reduction of the fungal indoor spores (by 20-fold) could be further achieved by the installation of HEPA filters.

### Sanitation and Cleaning Procedures

Besides air filtration, UV irradiation of the air or ozonation is recommended for the inactivation of airborne microorganisms in food processing environments (Varga and Szigeti, 2016). Implementation procedures, advantages and disadvantages of these methods have been reviewed (Masotti et al., 2019a). Inactivation of airborne microorganisms by ozonation and aerosolization with hydrogen peroxide has recently been tested in a dairy factory in Northern Italy (cheese making, storage, and packaging areas). The initial levels of yeasts in cheese-making facilities and packaging area (137 ± 439 CFU/m^3^) were eliminated by both treatments, most effectively by hydrogen peroxide (Masotti et al., 2019b). The occurrence of yeasts in dairy facilities suggests that regular monitoring of microbial counts, with subsequent cleaning procedures, should be implemented at dairies to reduce fungal contamination and to satisfy the GMP requirements. Cleaning and disinfection are specifically important in humid areas of the dairy production, such as wall corners, floor and floor drains, ceiling, and parts of equipment, where yeast species are able to survive and even proliferate (Awasti and Anand, 2020). Cleaning-in-Place (CIP) circular washing with different detergents and hot water is applied as a current standard GMP method of cleaning of tanks and piping in dairy industries, eliminating impurities and reducing the levels of microbial contamination (Memisi et al., 2015).

## Conclusion and Future Perspectives

From the present survey, it is clear that the very fast development within biotechnological methodologies for identification and detection of spoilage yeasts is only sparely reflected in the dairy industry. Especially, as the spoilage potential vary significantly between different species of spoilage yeasts, correct identification is of outmost importance. With an increased focus on food waste and extended shelf life of dairy products being exposed to increased storage temperatures, there is no doubt that control of spoilage microorganisms is more urgent than ever. Therefore, there is an urgent need for technologies that easily can be transferred to the food sector being affordable, with a high level of precision and easy to handle.

While molds are recognized as hazardous contaminants on the surface of solid and semi-solid surface-ripened cheeses, yeasts are due to their fermentative capacity recognized as the main spoilage microorganisms of white-brined cheeses. Even though a huge variety of yeast species have been identified from these types of cheeses, it is worth noticing that only a limited number of yeast species will be able to proliferate in the products, and that there is a clear link between contaminants with spoilage potential and product specifications. The most predominant yeast species causing quality defects seem to be the ones normally associated with dairy products, e.g., *D. hansenii*, *K. marxianus*, *K. lactis*, *Y. lipolytica*, and *G. candidum*. Due to its halophilic nature, the marine associated yeast species *D. hansenii* might often be present in brined dairy products being introduced with the NaCl (Haastrup et al., 2018). Even though this yeast species thoroughly has been reported to have beneficial effects for surface ripened red smear cheeses (Gori et al., 2007; Ryssel et al., 2015; Haastrup et al., 2018), its role in white-brined cheeses is not really understood. For yeast species as *K. marxianus* and, especially, *K. lactis*, gas production seems to be a significant problem due to their ability to ferment lactose and/or galactose, which have not been assimilated by the initial starter cultures. Also, other fermentable carbohydrates added together with ingredients, e.g., spices and vegetables, might enhance the growth of spoilage yeasts. Off-flavors are often linked to yeast species, having high enzymatic activities, such as *D. hansenii*, *G. candidum*, *K. lactis*, and *Candida* spp. Pigmentation might additionally occur due to the production of pyomelanins by *Y. lipolytica.* Even within these yeast species, significant intraspecies variations occur and the risk of persistent yeast strains, being able to establish in the production plant, should always be considered. Accumulation of BA due to the activity of *D. hansenii*, *Y. lipolytica*, *K. marxianus* and *S. cerevisiae* and other microorganisms might rise safety concerns during prolonged storage of the white-brined cheeses. The role of specific yeast strains, originated from varieties of white-brined cheeses, in production of BA is not clear and needs to be further investigated in relationship to the processing and storage conditions.

Despite the relatively simple processing technology, write-brined cheeses might globally be produced within many different flow charts, slightly differentiating them from each other. However, even small changes might have an impact on the extrinsic and intrinsic parameters and, thereby, on the yeast species being able to proliferate in this particular type of white-brined cheese. As a consequence, most producers of white-brine cheeses are not aware of the yeast species being the most harmful for their particular type of cheese, as well as the defects they might cause. Unfortunately, research within this scientific field is lagging far behind. Practical knowledge exists on the use of bio-protective cultures for white-brined cheeses; however, scientific knowledge on the interaction mechanisms is still missing, especially on the production of antifungal peptides for inhibition of spoilage yeasts. Likewise, other microbial interaction mechanisms and their influence on the proliferation of spoilage yeasts in white brined cheeses are not investigated in detail, i.e., it is unknown how the primary starter cultures and their lysis influence the growth of spoilage yeasts. As alternatives to costly bio-protective cultures, the possibility of development of new types of starter cultures, having potential to inhibit spoilage yeasts, should be explored. Implementation of good manufacturing practices is an integral part of the entire food chain, being a very important tool to prevent food waste and to optimize income generation. The careful design of processing plants should also be seen as an absolute requirement for safe food processing. Prevention of airborne contamination is often a neglected area, when it comes to yeasts. However, it is clear that yeasts can be transported in dairy plants through small aerosols. In this case, airborne yeasts need to be identified in order to evaluate their spoilage potential. Often yeast species in air samples are not capable of growing in the cheese, though their presence should still be avoided.

In conclusion, white-brined cheeses are globally produced in a variety of brands; even more diverse are the yeast species capable of causing quality defects of these cheeses. Unfortunately, detailed knowledge on spoilage potential and variations at species and strain level is still missing. Implementation of advanced technologies for species and strain identification, being simple to handle and of low cost, is still important to prevent food waste and enhance the sustainability of these types of cheeses. Gathering of scientific knowledge on yeast interactions with other dairy-related microorganisms as well as the cheese matrix, will add to an optimized production of white-brined cheese of enhanced quality.

## Author Contributions

LJ and AG designed the manuscript. AG, TS, GT, NL, and LJ wrote the manuscript. KR critically revised the manuscript. All authors contributed to the article and approved the submitted version.

## Conflict of Interest

GT is employed by the company Jotis S.A. The remaining authors declare that the research was conducted in the absence of any commercial or financial relationships that could be construed as a potential conflict of interest.
